# Contamination of SARS-CoV-2 in patient surroundings and on personal protective equipment in a non-ICU isolation ward for COVID-19 patients with prolonged PCR positive status

**DOI:** 10.1186/s13756-020-00839-x

**Published:** 2020-10-29

**Authors:** Li Wei, Wenzhi Huang, Xiaojun Lu, Yantong Wang, Linzhi Cheng, Rong Deng, Haiyan Long, Zhiyong Zong

**Affiliations:** 1 Department of Infection Control, West China Hospital, Sichuan University, Chengdu, China; 2 Department of Laboratory Medicine, West China Hospital, Sichuan University, Chengdu, China; 3 Center for Pathogen Research, West China Hospital, Sichuan University, Chengdu, China; 4 Center of Infectious Diseases, West China Hospital, Sichuan University, Chengdu, China

**Keywords:** Corona virus disease 2019, COVID-19, SARS-CoV-2, Air sampling, Environment sampling, Healthcare workers, Personal protection equipment

## Abstract

**Objectives:**

We performed an environmental sampling study to investigate the environmental contamination of SARS-CoV-2 by COVID-19 patients with prolonged PCR positive status of clinical samples.

**Methods:**

We sampled the air from rooms for nine COVID-19 patients with illness or positive PCR > 30 days, before and after nasopharyngeal/oropharyngeal swabbing and before and after nebulization treatment. We also sampled patients’ surroundings and healthcare workers’ personal protection equipment (PPE) in a non-ICU ward. SARS-CoV-2 was detected by PCR.

**Results:**

Eighty-eight samples were collected from high-touch surfaces and floors in patient rooms and toilets, with only the bedsheets of two patients and one toilet positive for SARS-CoV-2. All air samples (n = 34) were negative for SARS-CoV-2. Fifty-five samples collected from PPE were all negative.

**Conclusion:**

Contamination of near-patient surroundings was uncommon for COVID-19 patients with prolonged PCR positive status if environmental cleaning/disinfection were performed rigorously. Airborne transmission of SARS-CoV-2 was unlikely in these non-ICU settings.

Corona virus disease 2019 (COVID-19) caused by SARS-CoV-2 [1] has emerged as a global pandemic [2]. Patient surroundings have been suggested as a potential reservoir of transmission [3]. In our hospital, some non-severe COVID-19 patients had been hospitalized for > 1 month but still provided clinical samples positive for SARS-CoV-2 RNA, suggesting prolonged viral carriage. It is possible that COVID-19 patients with prolonged PCR positive status could continuously shed virus, contaminating their surroundings and therefore pose a substantial risk to healthcare workers (HCWs).

We performed two-round environment sampling in an isolation ward dedicated for laboratory-confirmed COVID-19 patients on March 4 and 12, 2020, respectively. On March 4, nine laboratory-confirmed COVID-19 patients were hospitalized in six rooms in a dedicated non-ICU isolation ward. Patients of the same family were placed in the same two-bed room; otherwise, patients. were placed in single rooms. On March 4, all 9 patients had been ill for > 30 days, while five remained hospitalized in four rooms on March 12 (Table 1). All patients exhibited mild COVID-19 using clinical criteria [4]. On March 4, all clinical samples (nasopharyngeal swab, oropharyngeal swab, sputum and stool) from four patients (patients A, B, D and E) were negative for SARS-CoV-2 as tested by real-time reverse transcriptase-polymerase chain reaction (RT-PCR, see below), while the other five patients (C, F, G, H and I) had one or two SARS-CoV-2-positive clinical samples, with two (patient G and I) asymptomatic (Table 1). On March 12, all 5 patients had symptoms and had one or two SARS-CoV-2-positive clinical samples (Table 1). Patient rooms are not under negative air pressure and there is a toilet for each room. As part of the routine care, clinical samples were collected regularly at intervals of two or three days with nasopharyngeal and oropharyngeal swabs and sputum samples collected in patient rooms and stool samples collected in toilets. Environment sampling was scheduled on the day of collection of clinical samples. This study was approved by the Ethics Committee of West China Hospital with oral informed consent being obtained.

**Table 1:** Sampling time points in relation to patient illness and results of clinical samples

Room	Patient	On the day of environment sampling				Most recent clinical samples (Ct values, ORF1ab/N genes)^b^
		Days of illness or positive PCR^a^	Symptoms	Nebuli- zation	Positive clinical samples (Ct values, ORF1ab/N genes)^c^	
1	A	33	None	+	All negative	Nasopharyngeal swab (36.5/27.7), day 31
2	B	34	Breath shortness, chest pain		All negative	Nasopharyngeal swab (36.5/19.4) & sputum (36.7/26), day 32
3	C	42	Cough	+	Nasopharyngeal swab (32.3/35.8)	
		50	Cough, fever		Nasopharyngeal swab (-/35.5) & sputum (21.4/24.9)	
4	D	39	Cough, sputum production, fever	+	All negative	Nasopharyngeal swab (28/29.3), day 37
		47	Cough, sputum production	+	Nasopharyngeal swab (-/37.3)	
	E	40	None		All negative	Oropharyngeal swab (25.1/23.5), day 38
5	F	44	Cough, sputum production	+	Sputum (28.0/20.8)	
		52	Cough, sputum production		Oropharyngeal swab (-/34.2) & sputum (23.4/25.1)	
	G	33	None		Nasopharyngeal swab (37.3/34.7)	
6	H	34	Cough, sputum production		Sputum (37.1/32.8)	
		42	Cough, sputum production		Nasopharyngeal swab (28.0/28.3)	
	I	35	None		Stool (37.9/26.0)	
		43	Palpitation, chest burning sensation	+	Nasopharyngeal (-/36.1) & oropharyngeal swabs (28.2/25.3)	

^a^For those without symptoms, the days refer to the positive PCR status

^b^On the day of environment sampling, nasopharyngeal swab, oropharyngeal swab, sputum and stool were collected for all patients with symptoms, while nasopharyngeal swab, oropharyngeal swab and stool were collected for all patients without symptoms. Cycle threshold (Ct) values of PCR were shown. -, negative at the 40th cycles

^c^The most recent positive clinical samples are shown for patients with no positive clinical samples on the day of environment sampling

First, we sampled air to detect the presence of SARS-CoV-2 in patient rooms, toilets, and a negative pressure room (12 air exchanges per hour) used specifically for interferon-α nebulization treatment. In patient rooms, air sampling was performed at a time point before any medical activities in the morning and at another point 15 min after nasopharyngeal swab and oropharyngeal swab collection from the patient or from the first patient if there were two in the room, which usually took one or two minutes for each swab. Air sampling was performed using an air microbiological sampler (FSC-1V; Hongrui, Suzhou, China) with 0.22 μm filter membranes on a nutrient agar (Hopebiol; Qingdao, China) plate for 15 min at 100 L/min, which was placed about 2 m away from patient and 1.1 m above the ground. In toilets, air sampling was performed in the morning with the sampler placed in the center of the room, 0.2 m above ground. Air was also sampled before and after performing nebulization treatment for all patients required (n = 4 on March 4 and n = 2 on March 12, Table 1). After air sampling, the filters and the surface of agar were wiped using sterile swabs (Copan; Brescia, Italy) premoistened with viral transportation solution (Longsee; Guangzhou, China). RT-PCR targeting the open reading frames 1a/1b (ORF1ab) and the nucleocapsid protein (N) gene was used to detect SARS-CoV-2 [5]. All air samples (n = 34) were SARS-CoV-2-negative. SARS-CoV-2 was not detected at distances > 1 m away from patients with a SARS-CoV-2-positive swab (n = 5 for nasopharyngeal and n = 2 for oropharyngeal) when collecting swabs nor after nebulization for patients with positive respiratory samples (Table 1). This suggests that nasopharyngeal/oropharyngeal swabbing and nebulization do not generate SARS-CoV-2-laden aerosols for long distance transmission.

Second, we sampled high-touch areas and floors in patient rooms and toilets, which were cleaned/disinfected twice daily. Before the first daily cleaning, high-touch areas in patient rooms (the entire surface of light switches, door handles, bed rails, bedside tables, tables on bed, equipment belts on wall, pillows, drinking bottle handles, and patients’ mobile phones, and 1,200 cm^2^ (30 cm × 40 cm) surface of bed linens, lockers, and the floor) and in toilets (the entire surface of light switches, door handles, handwashing sink rims, sink and toilet bowls and drains) were sampled using premoistened swabs (Copan). Eighty-eight samples were collected but only three samples (3.4%, 3/88) were SARS-CoV-2-positive (Table 2). The three SARS-CoV-2-positive samples were collected from the bed sheets of patients D and I (two different rooms) and the sink internal bowl and drain in the toilet of the room shared by patients H and I.

**Table 2:** Sampling results

Environmental sites	Number of positive samples/number of total samples (Ct values, ORF1ab/N genes)^a^		
	Room, patient D	Room, patients H/I	All patient rooms
*Patients rooms*			
1.Door handle	0/1	0/1	0/6
2.Table on bed	0/1	–	0/2
3.Bed sheet	1/3 (23.1/−^b^)	1/3 (32.9/30.7)	2/10
4.Bed rail	0/1	0/2	0/7
5.Bedside cabinet	0/1	0/2	0/9
6.Floor	–	–	0/2
7.Light switch	0/1	0/1	0/6
8.Surface of air purifier	0/1	0/1	0/4
9.Equipment belt on wall	0/1	0/1	0/4
10.Patient mobile phone	0/1	0/2	0/5
11.Thermos bottle handle	0/1	0/2	0/5
12.Locker	0/1	–	0/2
13.Pillow	0/2	0/2	0/6
*Toilet area*			
14.Door handle	0/1	0/1	0/5
15.Sink internal bowl and drain	0/1	0/2	0/5
16.Sink external rim	0/1	0/1	0/4
17.Toilet bowl and drain	0/1	1/1 (17.5/25.3)	1/6
Total, no. (%)	1/19 (5.3)	2/22 (9.1)	3/88 (3.4)

^a^Cycle threshold (Ct) values of PCR were shown for positive samples, while negative results refer to no amplification at the 40th cycles

^b^The N gene was able to be detected at the 42.2th cycle

It is notable that the contamination of SARS-CoV-2 in our isolation ward before routine cleaning was uncommon and was lower than that seen in previous studies (3.4% vs. 52.3 to 61% of all samples) [3, 6, 7]. There are several possible reasons. First, all patients had been hospitalized for > 1 month with mild symptoms when sampling and might have lower viral loads. Second, due to the mild disease, the activities performed by HCWs in the ward were simple with less interventional procedures that could generate respiratory aerosols or directly contaminate surroundings. Third, alcohol-based hand sanitizers were placed at each patient’s bedside, for each treatment cart and on the wall outside each patient room. All sheets and pillows were replaced, and toilets were flushed with 2000 mg/L chlorine solutions daily. The second-round sampling generated negative results, suggesting that such routine measures are effective.

Third, we sampled personal protective equipment (PPE) of eight HCWs. The entire outside surface of gloves, caps, N95 respirators or surgical masks, shoe covers, goggles, and face shields, and approximately 1050 cm^2^ (30 cm × 35 cm) outside surface of the isolation gowns and protective clothing were sampled using sterile premoistened rayon swabs (Copan) when the HCWs exited patient rooms. There were 55 samples collected from PPE and all were negative. It is interesting that shoe covers were negative, which could be due to the disinfection of the floor twice daily. All other PPE samples were also negative, consistent with a previous report [3], suggesting that PPEs worn by HCWs in this non-ICU isolation ward were not likely to be contaminated. The HCWs performed routine activities such as medical round, measuring vital signs, collecting clinical specimens of patients, performing intravenous infusion, and delivering food. HCWs in China usually chose to wear N95 respirators to prevent possible aerosols although they would not perform activities that have been proven to generate aerosols such as bronchoscopy, induction of sputum and non-invasive ventilation. The negative results of the surfaces of N95 respirators and face shields in this study suggests that at least at the later stage of COVID-19 SARS-CoV-2-containing aerosol and droplet generated by routine activities are absent or in low quantity and the route of air transmission for SARS-CoV-2 is unlikely.

We are aware of limitations of our study. We did not perform environment sampling at the early stage of COVID-19 when viral load in upper respiratory tract is higher for comparison [8]. We also did not perform viral culture to demonstrate viability. Furthermore, although we collected 1500 L air for one sample, it is a comparatively small volume compared to the entire space of the room. It is also possible that our air filter-based air sampling method is less sensitive than methods using liquid media [6]. We did not include negative and positive controls for sampling to demonstrate the detection limits, which should be considered in future studies.

In conclusion, COVID-19 patients could have prolonged (> 30 day) SARS-CoV-2 PCR positive status for clinical samples. For COVID-19 patients with prolonged viral carriage, the contamination of SARS-CoV-2 on patient surroundings is low, while air and HCWs’ PPE were not found to contain SARS-CoV-2. We do not find that nasopharyngeal/oropharyngeal swabbing and nebulization treatment generate aerosols able to transmit to long distance. Routine activities in non-ICU isolation wards are unlikely to generate SARS-CoV-2-containing aerosols and droplets. Our sampling results do not support the airborne transmission route of SARS-CoV-2 for patients with prolonged viral carriage.

## Data Availability

The data sets supporting the results of this article are included within the article.

## Abbreviations

COVID-19: Corona virus disease 2019
HCWs: Healthcare workers
PPE: Personal protective equipment
RT-PCR: Real-time reverse transcriptase-polymerase chain reaction
SARS-CoV-2: Severe acute respiratory syndrome coronavirus 2

## References

[1] Coronaviridae Study Group of the International Committee on Taxonomy of V. The species Severe acute respiratory syndrome-related coronavirus: classifying 2019-nCoV and naming it SARS-CoV-2. Nat Microbiol. 2020;5:536–44. 10.1038/s41564-020-0695-z PMC7095448 32123347

[2] Mahase E (2020). Covid-19: WHO declares pandemic because of "alarming levels" of spread, severity, and inaction. BMJ.

[3] Ong SWX, Tan YK, Chia PY, Lee TH, Ng OT, Wong MSY (2020). Air, surface environmental, and personal protective equipment contamination by severe acute respiratory syndrome coronavirus 2 (SARS-CoV-2) from a symptomatic patient. JAMA.

[4] National Health Commission of China. Guidance for the diagnosis and treatment of Corona virus disease 2019 Beijing, China; 2020.

[5] Li W, Ying B, Feng P, Kang Y, Huang Z, Song B (2020). A precision medicine approach to managing 2019 novel coronavirus pneumonia. Precision Clin Med.

[6] Zhou J, Otter JA, Price JR, Cimpeanu C, Garcia DM, Kinross J, et al. Investigating SARS-CoV-2 surface and air contamination in an acute healthcare setting during the peak of the COVID-19 pandemic in London. Clin Infect Dis. 2020:In press. doi:10.1093/cid/ciaa905. 10.1093/cid/ciaa905 PMC7454437 32634826

[7] Chia PY, Coleman KK, Tan YK, Ong SWX, Gum M, Lau SK (2020). Detection of air and surface contamination by SARS-CoV-2 in hospital rooms of infected patients. Nat Commun.

[8] Zou L, Ruan F, Huang M, Liang L, Huang H, Hong Z (2020). SARS-CoV-2 viral load in upper respiratory specimens of infected patients. N Engl J Med.

